# The Role of Purkinje-Myocardial Coupling during Ventricular Arrhythmia: A Modeling Study

**DOI:** 10.1371/journal.pone.0088000

**Published:** 2014-02-07

**Authors:** Elham Behradfar, Anders Nygren, Edward J. Vigmond

**Affiliations:** 1 Department of Electrical and Computer Engineering, University of Calgary, Calgary, Alberta, Canada; 2 Institut LIRYC and Laboratoire IMB, Universite Bordeaux 1, Bordeaux, France; Gent University, Belgium

## Abstract

The Purkinje system is the fast conduction network of the heart which couples to the myocardium at discrete sites called Purkinje-Myocyte Junctions (PMJs). However, the distribution and number of PMJs remains elusive, as does whether a particular PMJ is functional. We hypothesized that the Purkinje system plays a role during reentry and that the number of functional PMJs affect reentry dynamics. We used a computer finite element model of rabbit ventricles in which we varied the number of PMJs. Sustained, complex reentry was induced by applying an electric shock and the role of the Purkinje system in maintaining the arrhythmia was assessed by analyzing phase singularities, frequency of activation, and bidirectional propagation at PMJs. For larger junctional resistances, increasing PMJ density increased the mean firing rate in the Purkinje system, the percentage of successful retrograde conduction at PMJs, and the incidence of wave break on the epicardium. However, the mean firing of the ventricles was not affected. Furthermore, increasing PMJ density above 13/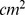 did not alter reentry dynamics. For lower junctional resistances, the trend was not as clear. We conclude that Purkinje system topology affects reentry dynamics and conditions which alter PMJ density can alter reentry dynamics.

## Introduction

The Purkinje System is the fast conduction system of the heart, responsible for ensuring coordinated contraction of the ventricles. Despite its importance, many important small details remain unknown, and these details affect electrical wave propagation at the whole organ level. Recording Purkinje system activity using multielectrode catheters has provided some insights into Purkinje system involvement in ventricular fibrillation (VF): Bidirectional propagation between Purkinje system and myocardium has been observed during VF in canine heart [1]; cryoablation of canine endocardium changed the activation pattern of VF [2]; and chemical ablation of Purkinje system led to faster termination of VF and lower activation rate in dogs [3]. However, the spatial resolution obtained by this technique is not sufficient for direct detection of electrical activity of the Purkinje system since its extremely fine structure prevents recording its activity over a large area. Furthermore, even methods which have successfully reconstructed the topology of the endocardial network have been unable to map the insertion of the Purkinje system into myocardium where it forms Purkinje-Myocyte Junctions (PMJs), which are responsible for activating the ventricular muscle. Finally, even if the PMJs are imaged, be it by histology or otherwise, their functional role still requires electrophysiological evaluation since evidence points to a large number of nonfunctional junctions [4]. To date, the number and distribution of functioning PMJs is unknown and no technique currently exists to directly measure it.

PMJ density may change due to increases in heart size, as during dilated cardiomyopathy (DCM) for example, or alterations in Purkinje system structure, for example, due to aging [5]. Ischemia has also been shown to alter the number of functional PMJs [6] as have changes in cellular coupling [4]. PMJ density was shown to have an effect in a model of biventricular pacing [7] but its effect on reentry dynamics are not known. More PMJs can lead to more retrograde conduction and allow more escape paths for arrhythmias but can also decrease tissue heterogeneity. Thus, its actions are both pro- and antiarrhythmic [8]. From a modeling perspective, given studies by our group using simplified Purkinje system representations[9]–[11], it is important to know if results are relevant given the true complicated Purkinje system structure.

This study hypothesized that PMJ density has an effect on reentry dynamics, and sought to determine the minimum density to incorporate into models. This was done by initiating fibrillation in a biventricular computer model and quantifying reentry characteristics as a function of PMJ density. We also performed a sensitivity analysis of the parameters used to control activity across the PMJ structure.

## Methods

### Purkinje Network Modeling

**Figure 1:**
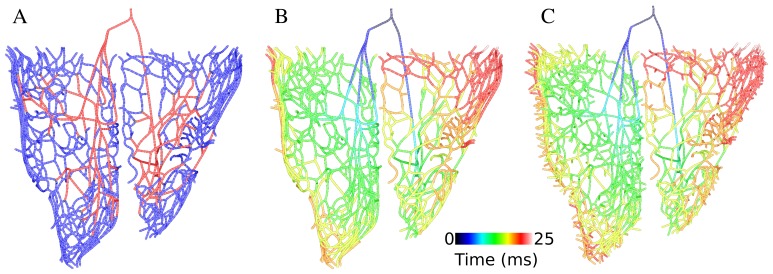
3D model of Purkinje network. A) new model of Purkinje network consists of manually constructed branches (red) and extended branches (blue) B) Purkinje system model with lowest PMJ density (74 PMJs) and its activation map for His bundle stimulation C) Purkinje system model with highest PMJ density (516 PMJs) and its isochronal activation map.

To develop an anatomically detailed model of Purkinje system, we used a fractal method [12] to extend the Purkinje network that had been manually constructed by our lab [13], shown in Fig. 1A in red. To grow new branches of Purkinje system on the endocardial surface, endpoints of the manually constructed network were used as starting points. The growth process was based on extended L-system and a generating rule suggested by Ijiri et al [12]. The parallel rewriting system replaces each part of the current structure by applying the rule sequentially and generates a uniform distribution of branches on the endocardial surface. Endocardial surfaces were extracted from an FEM model of the rabbit ventricles and the outcome was two triangularly meshed surfaces. Each new generated point was projected onto the closest triangle of the endocardial surface at each iteration, thereby ensuring the Purkinje system exactly lay on the endocardium.

Adjusting the parameters of L-system was based on comparisons with photographs of actual rabbit Purkinje system [14] and activation patterns in rabbit ventricles. The branch length was set to 2 mm and each branch consisted of five line segments to obtain a more realistic branch curvature. Fig. 1A illustrates extended branches connected to 53 endpoints of the manually constructed network.

The next step of constructing the Purkinje system model was to add the endpoints, which contact with ventricular myocytes and activate them through PMJs. In light of the dearth of data on PMJs, endpoints were uniformly distributed on extended branches of Purkinje system by setting a minimum distance between them and then inserting them into the myocardium up to 20% of wall thickness. To create various PMJ densities, seven different values for the minimum distance between Purkinje system endpoints were set. In the end, Purkinje system models with 74, 105, 166, 244, 325, 451 and 516 PMJs were generated. Fig. 1B and C shows the new Purkinje system models with different PMJ density and the activation sequence after His stimulation. The total activation time of Purkinje system was about 25 ms.

Mathematical details of the PMJ structure and its coupling to myocardium are found in [13]. In brief, at each PMJ, a Purkinje element was coupled to the 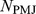 closest myocardial points by a fixed resistance 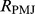. The current load on each terminal Purkinje cell was calculated by summing the individual currents flowing into each junctional myocyte and scaling the total by a loading factor 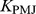 to account for myocardial loading effects from surrounding tissue. This implementation was designed to attain discontinuous and asymmetric propagation across the PMJ [15]. The sensitivity of transmission delay to changes of PMJ model parameters was evaluated and parameters were sought to match experimental measurement. Propagation delay at PMJs was defined as the difference between activation time of the terminal Purkinje node and the average activation time of coupled myocytes. We found a range of values of junctional parameters (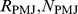) which resulted in realistic anterograde and retrograde delays. Anterograde delay times in the range of 4–14 msec and retrograde delay times between 2–4 msec were considered physiological [16], [17]. Combinations of 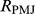 and 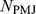 values resulting in realistic transmission characteristics were in the ranges 10–25 M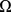 for 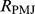, and 10–30 for 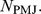


### VF Induction in Ventricle Model

Computer simulations were performed on an image based computer model of rabbit ventricles complete with Purkinje system as used extensively by our group[8]–[11], [13], [18]. Cardiac electrical activity was described by the bidomain equations [19] which relate the intracellular to the extracellular potential through transmembrane current density. The system of equations was solved by the finite element method using the Cardiac Arrhythmias Research Package (CARP) [20]. Ion dynamics for the myocardium were described by the rabbit ventricular action potential (AP) model developed by Mahajan et al. [21], while the Purkinje cell of Aslanidi et al. [22] was used for the Purkinje system.

Reentry was initiated through an S1–S2 protocol: for S1, two His paces were delivered 500 ms apart. S2 was an extracellular cross shock delivered by applying a uniform electric field via two planar electrodes at bath boundaries oriented along the plane of the ventricular septum. S2 followed S1 after a variable coupling interval (CI). The strength of the shock and the CI between S1 and S2 were varied to find combinations that induced sustained reentry, that which lasted for at least 3 s.

To better model responses to electric shocks, ionic models were modified to include an electroporation channel [23] and an outward current activated during shock-induced depolarization [24]. To facilitate VF induction, 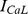 (L-type Ca current) recovery from inactivation kinetics was increased for the intermediate range of diastolic interval (DI) values (10–50 ms).

### Data Analysis

For assessing the role of the Purkinje system and Purkinje-myocardium coupling during arrhythmia, reentry dynamics were evaluated using three measures: 1) Mean firing rate (MFR) was quantified for every node of the tissue model, by inspecting membrane voltage (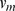) and counting activations by threshold crossing at −20 mV with a 50 ms blanking interval. MFR was calculated by dividing the number of activations by the duration of the associated counting interval which was 3 s here.

**Figure 2:**
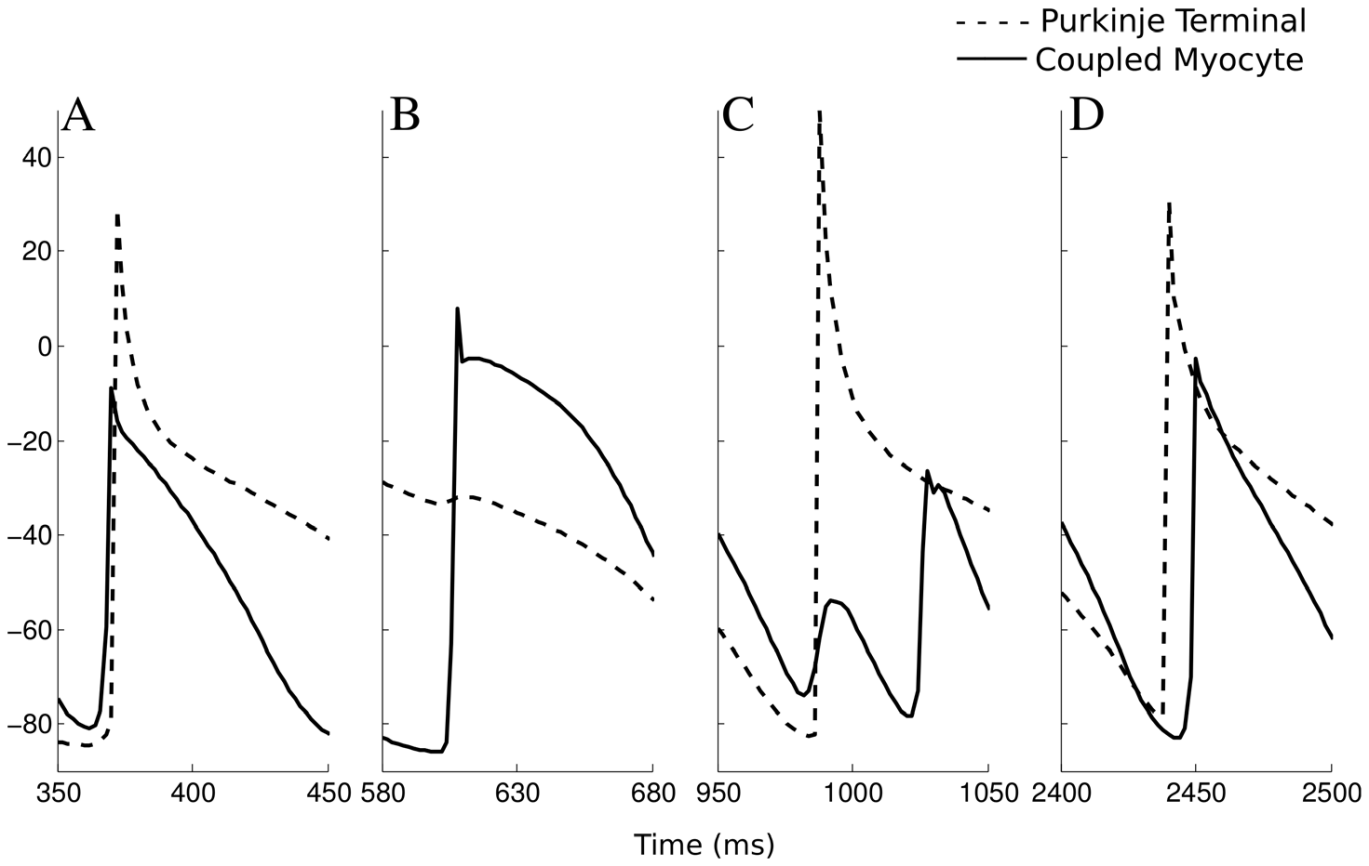
Instances of conduction success and failure at one PMJ. A) successful retrograde propagation, B) failed retrograde propagation C) failed anterograde propagation D) successful anterograde propagation.

2) A useful measure for evaluating spatial organization of VF is the number of phase singularities that form during VF. An algorithm to detect phase singularities was used which relied on the intersection of two isosurfaces which were computed at a certain temporal interval [25]. This reduces to finding points or lines along which the temporal derivative is zero. Filament analysis was carried out with the isosurface threshold set to −40 mV and the interval between slices set to 8 ms. We then calculated the WaveBreak Incidence (WBI) [26] by counting the phase singularities on the epicardium and endocardium surfaces separately, and normalizing by the surface area and the average number of excitation cycles estimated as the product of MFR and reentry duration. An increase in WBI indicates a more complex arrhythmia since there are more phase singularities. 3) We also counted the number of successful and failed anterograde and retrograde propagations during reentry in the whole model, which directly shows the contribution of the Purkinje system to the reentry circuit. A conduction at the junction was considered successful when the terminal Purkinje node and more than 50% of coupled myocytes activated within a reasonable time interval for anterograde and retrograde propagations, otherwise conduction at the PMJ was considered to have failed. The acceptable time interval was selected as 8 ms for retrograde propagation and 16 ms for the reverse direction. When 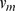 started below −60 mV and depolarized above −20 mV, it was counted as an AP. Fig. 2 illustrates examples of successful and failed retrograde and anterograde conductions for a PMJ during 3 s VF. PMJ parameters were chosen such that starting from the resting state, propagation was always successful. Failure occurred due to dynamical properties of the junction, most commonly that the the post junctional tissue was refractory, rapid activation possibly reducing available sodium current, or a combination of the two.

## Results

### Sinus Activation

**Figure 3:**
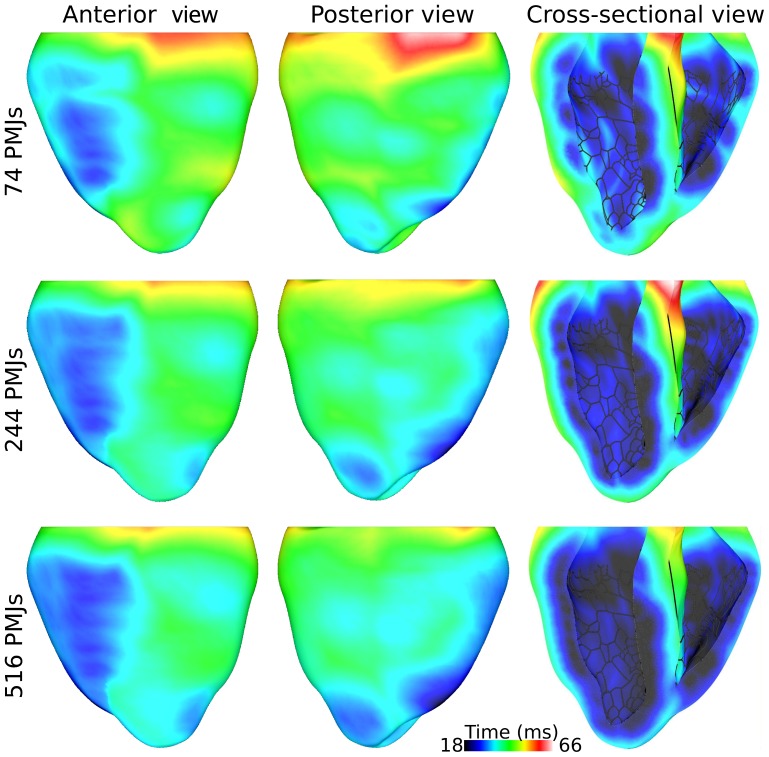
Activation maps for different PMJ densities. Excitation of the ventricular myocardium by the Purkinje system during sinus rhythm for Purkinje system models with three different numbers of PMJ as indicated on the left. Activation time since His bundle stimulation is scaled between earliest and last epicardial activation over all simulations.

The extended Purkinje system was superimposed on the endocardium of rabbit ventricles model and sinus rhythm was simulated by stimulating the His bundle. The resultant activation sequence for Purkinje systems with different PMJ densities were compared. The time of first breakthrough on epicardium varied from 18 to 26 ms which was inversely proportional to PMJ density. Fig. 3 illustrates the epicardial isochronal activation maps from the anterior and posterior perspectives. Activation in ventricles started from the medial or apical part of the right ventricular free wall and propagated toward the base of heart. In the left ventricle, the action potential broke through towards the septum and apex. Similarity, the activation patterns for the three PMJ densities depicted in Fig. 3 suggest that the number of uniformly distributed PMJs does not considerably affect the sequence of ventricular activation. For the model with 74 PMJs, the ventricles were entirely activated after 66 ms, and with 516 PMJs, this time reduced to 58 ms.

Right and left basal regions excited at the end of this interval. Epicardial activation sequences were compared to those obtained from ventricular epicardial unipolar electrograms and optical mapping of a rabbit heart during sinus rhythm [27], [28]. The early epicardial breakthrough sites reported in theses experimental studies are right ventricle freewall and left ventricle apex. Activation spread generally from apex to base and finally to the left ventricle base. Although the total activation time measured experimentally is shorter than what we found, the predicted activation sequence using our model was similar to the experimentally measured activation sequence.

### Effect of PMJ Density

The earliest propagated post-shock activation which initiated reentry originated from the Purkinje system in all simulations. To clarify the role of the Purkinje system and especially Purkinje-myocardium coupling during maintenance of reentry, we studied reentry patterns 2 s after initiation of reentry. At this stage, the transient have died down and a more stable reentry has been established.

**Figure 4:**
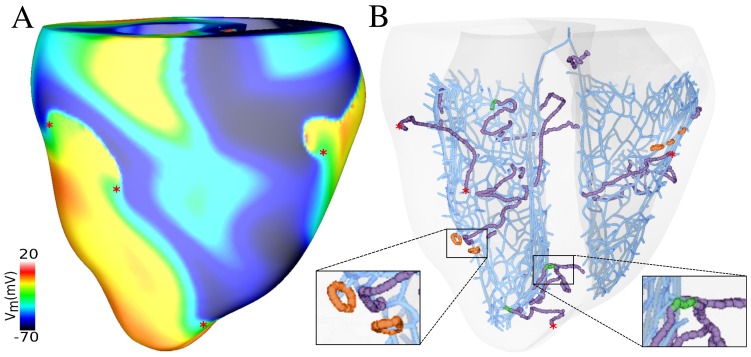
Purkinje system contribution during reentry. A) Rotors on the epicardium surface along with phase singularities indicated by red asterisks. B) Filaments in ventricles colocalized with Purkinje system endpoints. I-shaped filaments which terminate close to Purkinje system endpoints are shown in green, and O-shaped filaments which formed close to Purkinje system endpoints are indicated in orange. Surface phase singularities are as in A.

Fig. 4A shows a snapshot of VF generated by sustained reentry along with phase singularities detected on the epicardium. The complicated patterns of VF were driven by simultaneous sources of functional reentry. The Purkinje system favors reentry by providing pathways through retrograde and anterograde conduction at PMJs However Purkinje system involvement in reentry may stop propagation of excitation in certain direction or fractionate reentrant wavefronts due to refractory regions surrounding PMJs, which consequently increased the complexity of excitation dynamics. These antagonistic effects have been described previously [8], [18].

As can be seen in Fig. 2, the activation pattern at a particular PMJ had little consistency over time and bidirectional conduction and block occurred, indicative of functional reentry. Filaments in the ventricles are displayed in Fig. 4B which shows most of the wavebreaks were associated with Purkinje system activity. Most of the transmural I-shaped filaments were anchored to Purkinje system endpoints and O-shaped filaments formed close to the Purkinje system endpoints.

**Figure 5:**
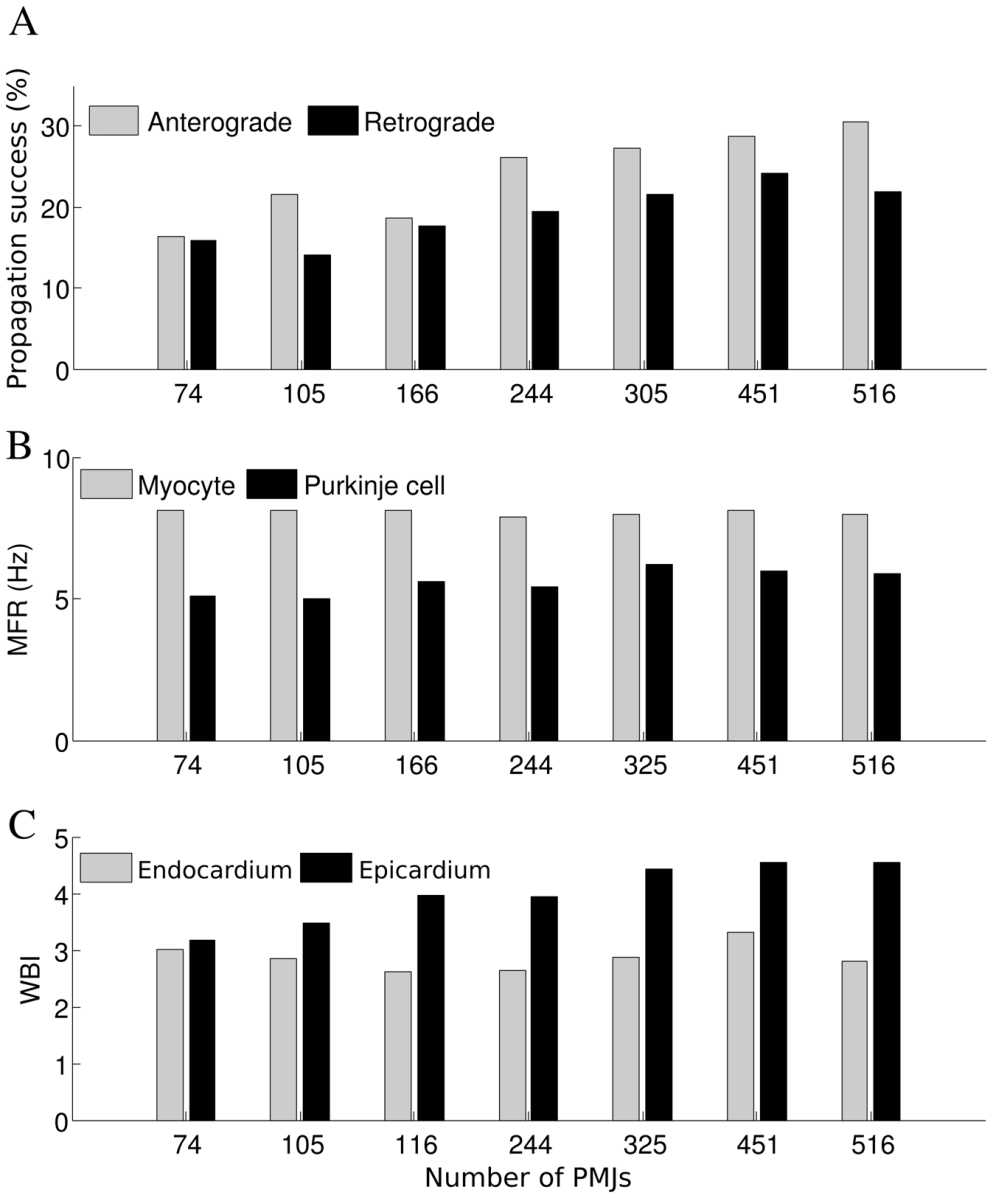
Changes in reentry dynamics as a function of PMJ density. A) Portion of successful junctional propagations as a function of PMJ density B) Average MFR in ventricles and Purkinje system as a function of PMJ density C) WBI on epicardium and endocardium as a function of PMJ density (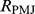 = 20 M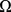, 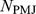 = 20).

Fig. 5 illustrates how changes in PMJ density affected reentry. The number of failed and successful propagations increased monotonically as the density of PMJs in the model increases, either for retrograde or anterograde transition. The portion of successful propagations for different PMJ densities are shown in Fig. 5. In models with higher numbers of PMJs, the contribution of the Purkinje system increased. More successful bidirectional propagation at PMJs showed a more significant role of the Purkinje system in establishing the reentrant pathway,and a better synchronization of activity. Generally, anterograde propagation was more successful than retrograde since the myocardium had a shorter APD and higher MFR, lessening chances of myocardial tissue being recovered when activity tried to emerge from the PMJ.

The number of successful retrograde transmissions of APs to the Purkinje system is an indicator of the degree to which ventricular activity drives Purkinje system activity; thus the increase of MFR in the Purkinje system was linked to the number of retrograde propagations. This can explain why the MFR in the Purkinje system is higher for larger number of PMJs (Fig. 5B). Average MFR in the ventricular mass changed in the narrow range of 7.8–8.2 Hz and did not show a sensitivity to junctional parameters or PMJ density Although there was a direct relation between the number of successful anterograde propagations and the average MFR in ventricles, changes in ventricular MFR were not considerable. In the cases that reentry was not sustained and lasted for less than 3 s, the number of successful anterograde propagations, the average MFR in ventricles, and the WBI were all lower compared to sustained reentry which demonstrates the interplay among these three parameters.

An increase in successful anterograde propagation corresponded to a higher WBI on the epicardium, while the number of successful retrograde transmissions closely followed the endocardial WBI (see Fig. 5C). Successful anterograde propagation could initiate a new epicardial breakthrough, leading to the collision of the new wave with existing meandering wave fronts, causing a new wavebreak. When anterograde propagation was unsuccessful, activation did not spread in the ventricles and refractoriness around the Purkinje system endpoint was prolonged, potentially leading to more wave front fractionation, generating more wavebreaks. A higher WBI on the endocardium signified more wavefronts and, hence, more chance for a wavefront to find a recovered PMJ to enter. This follows since the Purkinje system is a rapidly conducting cable network which implies that cells over a large region are in a similar phase. With more wavebreaks, the myocardium displays more phases over a smaller region and the odds of finding the proper combination of Purkinje system and myocardial phases for retrograde reentry are greater.

### Effect of Junctional Characteristics

We also conducted a set of simulations to study how junctional characteristics altered the interaction between the Purkinje system and ventricular tissue during fibrillation with three different PMJ numbers: 105, 244 and 455.

During sinus rhythm, the average delay time between the activation of a terminal Purkinje system cell and coupled myocytes was calculated and sensitivity of latency to resistance and size of the junction were investigated. We found that variations in junctional resistance were directly related to changes in forward and reverse transmission delays at the PMJ. An increase in the size of the junctions (number of coupled myocytes) shortened anterograde and retrograde delays. Reduction of the transmission delay for anterograde and retrograde propagation is the result of decreased electrical loading at the junction which increases the safety factor for propagation.

**Figure 6:**
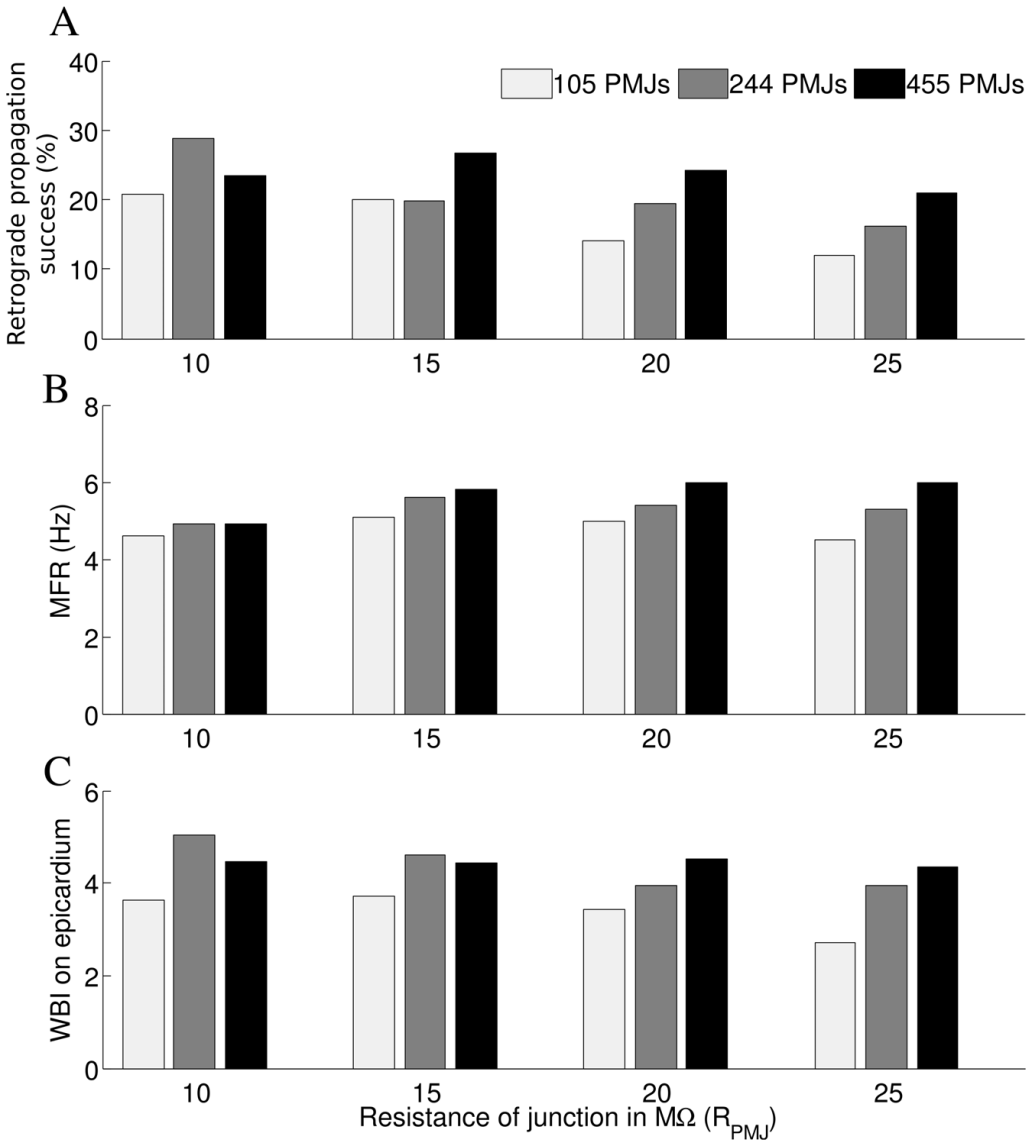
Effects of PMJ resistance on reentry. A) Ratio of successful retrograde propagations to failed and successful retrograde propagations as a function of PMJ resistance B) Sensitivity of average Mean Firing Rate in Purkinje system to the resistance of PMJ C) Sensitivity of WBI on epicardium to resistance of PMJ (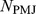 = 20).

We assessed the consequences of changes in resistance of PMJ (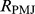) on reentry dynamics. For a larger PMJ resistance, there were generally fewer retrograde propagations (Fig. 6A and B), and the effect was greater for fewer junctions. Increasing the resistance tended to increase the MFR for the denser Purkinje system networks, while WBI was decreased.

**Figure 7:**
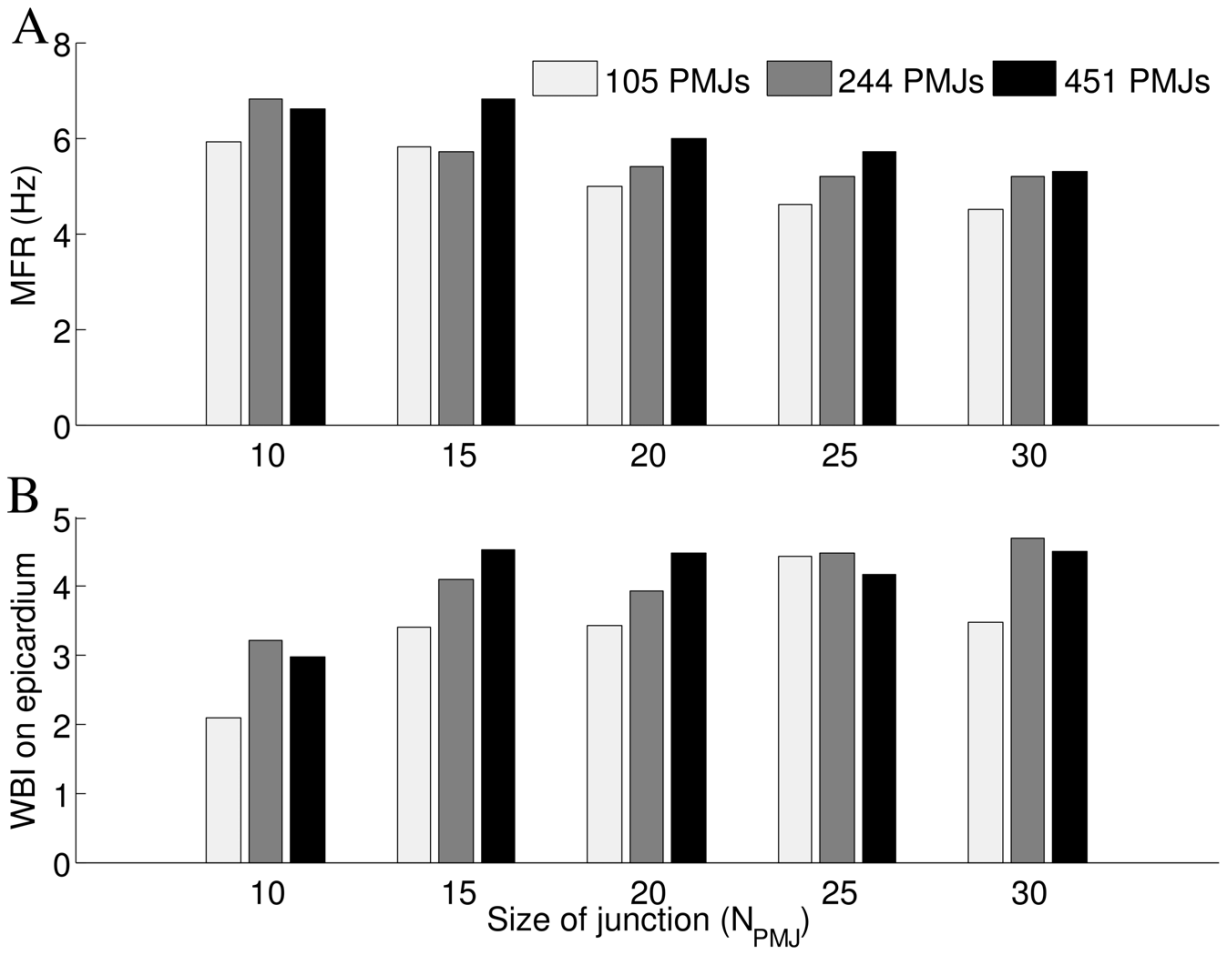
Effects of PMJ size on reentr. A) Sensitivity of average MFR in Purkinje system to the size of PMJ B) Sensitivity of WBI on epicardium to size of PMJ (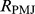 = 20 M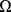).

Effects of PMJ size, 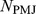, on reentry dynamics were investigated by calculating MFR, WBI and the number of propagations at PMJ. For both forward and reverse propagation at the PMJ, as the PMJ enlarges, the number of failed propagations decreased more than successful ones which means that the transmission of action potentials at the PMJ was easier. Fig. 7 demonstrates that for a larger PMJ size, there is a lower MFR in the Purkinje system. In spite of lower values of MFR in the Purkinje system, the number of successful anterograde propagations does not decrease with an increase of junction size, implying that the Purkinje system more successfully activates the myocardium after the increase in size of the junction and as a result, the WBI is higher (Fig. 7B).

## Discussion

We have implemented a fractal method for growing the Purkinje system to allow investigation of PMJ density on reentry dynamics, something which has not been previously explored. We varied coupling parameters over a wide range, but stayed within physiological bounds as determined by transmission delays. Our simulations are the first to gauge the contribution of the Purkinje system to reentry during shock-induced VF. We are able to conclude that there is a greater effect with a higher PMJ density. Comparing a variety of quantitative metrics, the dynamics of sustained reentrant arrhythmias were found to vary marginally for different coupling strengths at PMJs.

In spite of many efforts to extract Purkinje network from imaging data, it has not been extensively modeled in detail, due to its intricate structure and subendocardial penetration. Realistic geometrical models of the free running specialized conduction system have been constructed. In these studies, Purkinje system structure has been extracted from segmentation of MRI images [28], or by immunoenzyme labeling of a marker protein [14], [29]. As an alternative, like in this work, fractals have been used to build 3D anatomical models of the His€Purkinje network through automatic or semiautomatic procedures [12], [28]–[30]. Our Purkinje system model was designed in two steps: first, main fascicles and branches were created manually based on schematics and pictures of the conduction system and excitation mappings of the heart [13]. Second, detailed structures and endpoints generated by rewriting rules automatically. Since endpoints were only added to the extended branches of Purkinje system model and not to the manually constructed branches, the ventricular activation sequence with extended Purkinje system is similar to that with manual Purkinje system model. Also as reported in experimental studies [14], the Purkinje system in the left ventricle is more developed than that in the right ventricle, probably a result of function, and our extended model contains more branches in the left ventricle. There is limited knowledge about the number and distribution of PMJs in ventricular tissue but it is found that there are not any junctions in basal and top portion of septal regions of ventricles in different species [29]. We took these features into account to construct a Purkinje system model which replicates known characteristics of the junctions.

### Effect of PMJ Density

Our rabbit model varied the number of PMJs from 104 to 516 Given an endocardial surface area of 16.57 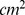, this corresponds to densities ranging from 4.46 to 31.14 PMJs/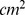. Scaled to a human heart, this would implicate 1000 to 5000 PMJs. With more PMJs, the activations sequence did not change considerably, but the time to activate was reduced. Part of the reduced time could be a consequence of the Purkinje system extending farther toward the base to accommodate more PMJs.

PMJ density did not affect the mean myocyte firing frequency during VF which was constant at 8 Hz. However, the Purkinje system MFR did increase, albeit slightly, with the number of PMJs, but only up to 325 junctions. This roughly corresponds to the trend in the percentage of successful propagations at the junction which generally increased with the number of PMJs. The limit of 325 also agrees well the the epicardial WBI dependence on PMJs. Since Purkinje system APs are intrinsically longer than ventricular ones, the upper limit of the Purkinje system MFR is lower than that of the ventricles. However, if the activity is better synchronized between the Purkinje system and ventricles, more successful junctional transmissions will occur and drive the Purkinje system MFR towards the maximum.

Interestingly, the endocardial WBI slightly decreased with higher PMJ density. The behavior most closely followed the ratio of retrograde success. Successful retrograde propagation would mean that endocardial wavefronts would reenter the Purkinje system and not interact with a refractory PMJ, possibly otherwise resulting in wave fractionation and an increase in WBI. Since the WBI was higher on the epicardium, the filaments could not all be transmural I-filaments since this would imply an equal endo- and epi-cardial WBI. Instead some U-filaments with both termini on the epicardium must have been present.

### Effect of Purkinje-myocardium Coupling

We have used a continuum model although at a fine scale this does not hold. The Purkinje system begins as a bundle of fibres which do not bifurcate. Whether or not Purkinje fibres do bifurcate and, if so, at which point is not known. At the PMJ, we have resorted to a phenomenological approach to incorporate asymmetric transmission. The PMJ radius corresponds to the terminal “branching” of the Purkinje system and the size of the region stimulated by concurrent Purkinje system activity. A greater radius means more myocardial cells are simultaneously activated and they may be thought of as a functional unit.

The coupling resistance is another important parameter. The range of values supporting propagation across the junction is actually quite limited since too large of a value does not allow enough charge to pass quickly enough to charge the postjunctional membrane, and too low of a value leads to excessive electrotonic loading which suppresses prejunctional action potentials.

The Purkinje system involvement in VF strongly depends on Purkinje-myocardium coupling as it affects bidirectional propagation at PMJs. We counted the number of successful and failed propagations at PMJs for both bidirectional conductions during reentry. The ratio of conduction success for anterograde propagation was higher than that for retrograde propagation. This can be attributed to the longer refractory period of Purkinje cells and almost simultaneous activation of close PMJs due to rapid conduction in the Purkinje system.

For a higher PMJ density, the activation wavefront has a higher chance of finding a pathway through the Purkinje system which increases the ratio of bidirectional conduction success and is favorable to maintenance of reentry. The results summarized in Fig. 5 suggest that number of phase singularities change proportionally with PMJ density. We attribute these changes to the interactions of filaments with Purkinje system endpoints. Previous modeling studies also suggested the presence of phase singularities around the distal Purkinje system network [18].

In the presented model of the PMJ, the increase of electrical loading through increasing junctional resistance 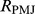 or reduction of junctional size 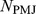, increases transmission delay across the PMJ for both directions. Sensitivity of anterograde delay to junctional parameters is more significant than that for retrograde conduction. Also propagation delay varies considerably more for changes of 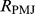 rather than changes of 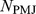.

The impact of transmission delay at junction on reentry dynamics is not straightforward to assess. While reduction of the transmission delay causes safer conduction and provides a pathway for reentrant wavefronts, slow conduction through the PMJs can be arrhythmogenic as it may assist survival due to increased effective path length.

The average ventricular MFR was unaffected by Purkinje system parameters and similar to the dominant frequency (DF) of ECG reported in experimental studies (average > 8 Hz) for VF in healthy rabbit heart [31], and higher than similar modeling study without Purkinje system (average 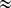 6 Hz) [32]. This is in agreement with reported simulation results for ventricular model with and without Purkinje system [18]. This suggests that the Purkinje system serves to reduce the reentry pathlength to the minimum one, which depends on the ventricular refractory period. The overall complexity of reentry was mostly governed by cardiac wavelength and ventricular size and geometry, and does not appear to be significantly affected by Purkinje system complexity.

### Implications for Pathophysiology and Aging

Experimentally it was found that aging is associated with functional and topological changes in Purkinje system [5]. These include thinning of Purkinje fibers and a decreased number of connections. This in itself is probably antiarrhythmic since it will reduce the probability of a afterdepolarization arising in the Purkinje system, without compromising activation or increasing complexity of any arrhythmias which arise. However, to investigate aging effects on ventricular arrhythmia, other age-related changes such as increase in ventricular stiffness and wall thickness, and electrical remodeling must be considered.

Another application of our study is assessing Purkinje system contribution to arrhythmias in pathological conditions that lead to ventricular remodeling such as dilated cardiomyopathy (DCM) and hypertrophic cardiomyopathy (HCM). Previously DCM and HCM have been modeled by increased wall thickness and increased ventricular diameter, respectively [7]. Assuming that Purkinje system branches and PMJs are not affected in ventricular remodeling, it was suggested that HCM has a higher than usual PMJ density due to a smaller endocardial surface while the DCM has a lower density due to larger endocardial surface. By varying the number of PMJs, we also implemented these changes in PMJ density, but also studied the consequences on reentry dynamics. For example, clinical reports indicate that occurrence of lethal ventricular tachyarrhythmias in patients with HCM reduces with age, and in old patients, mortality is unassociated with HCM [33]. A possible factor is the reduction of the number of connections between the Purkinje system and myocardial tissue with age, which will make reentry simpler.

Although in this study we set junctional parameters so that transmission delay across PMJs are within physiological range [16], [17], arrhythmogenic effects of drugs which manipulate junctional coupling at PMJs can be investigated using the same modeling scheme. An example is octanol which increases gap junctional resistance. In a study on canine subendocardial Purkinje to ventricular transmission, it was revealed that octanol causes a selective delay across PMJs which is believed to be mainly result of increase in junctional resistance [34]. Other diseases that affect cellular coupling, such as through changes in gap junction expression or fibrosis, will also affect the number of functional PMJs [4].

### Study Limitations

A shortcoming of our model is the simplification in modeling of PMJs structure and distribution which is a result of limited quantitative data available on the morphology and functioning of the junctions. The model does not explicitly incorporate transitional cells which couple Purkinje cells to ventricular myocytes [15], [35]. Nonetheless, due to the design of the model with its asymmetrical loading and source effects, it reproduces retrograde and anterograde transmission features, recreates measured junctional action potential traces, and effectively excites the ventricles. Also PMJs were distributed uniformly on the branches of Purkinje system while the location of PMJs on the subendocardial layer has not been identified.

In addition, validation of the model parameters and results based on results from experimental studies on rabbit heart may not be always practicable due to lack of experimental data, for instance, recording of Purkinje system electrical activity during arrhythmia has practical limitations which restricts us to quantify the accuracy of the model. Also VF mostly occurs in diseased hearts, whereas we simulated VF in healthy rabbit heart and VF mechanisms may be affected by abnormalities in cardiac tissue.

One issue not addressed is the increased propensity for afterdepolarizations with an increased Purkinje system. With more PMJs and more arborizations, there would be more Purkinje cells susceptible to afterdepolarization generation. The increase in afterdepolarization probability would be proportional to the length of the Purkinje system.

## References

[1] Tabereaux PB, Walcott GP, Rogers JM, Kim J, Dosdall DJ, et al (2007) Activation patterns of Purkinje fibers during long-duration ventricular fibrillation in an isolated canine heart model. Circulation 116: 1113–9. 17698730 10.1161/CIRCULATIONAHA.107.699264

[2] Janse MJ, Wilms-Schopman FJ, Coronel R (1995) Ventricular fibrillation is not always due to multiple wavelet reentry. Journal of cardiovascular electrophysiology 6: 512–21. 8528486 10.1111/j.1540-8167.1995.tb00424.x

[3] Dosdall DJ, Tabereaux PB, Kim JJ, Walcott GP, Rogers JM, et al (2008) Chemical ablation of the Purkinje system causes early termination and activation rate slowing of long-duration ventricular fibrillation in dogs. American journal of physiology Heart and circulatory physiology 295: H883–9. 18586887 10.1152/ajpheart.00466.2008 PMC2519198

[4] Morley GE, Danik SB, Bernstein S, Sun Y, Rosner G, et al (2005) Reduced intercellular coupling leads to paradoxical propagation across the Purkinje-ventricular junction and aberrant myocardial activation. Proc Natl Acad Sci U S A 102: 4126–4129. 15753312 10.1073/pnas.0500881102 PMC554832

[5] Cooper LL, Odening KE, Hwang MS, Chaves L, Schofield L, et al (2012) Electromechanical and structural alterations in the aging rabbit heart and aorta. American journal of physiology Heart and circulatory physiology 302: H1625–35. 22307668 10.1152/ajpheart.00960.2011 PMC4747897

[6] Kienzle MG, Tan RC, Ramza BM, Young ML, Joyner RW (1987) Alterations in endocardial activation of the canine papillary muscle early and late after myocardial infarction. Circulation 76: 860–874. 3652425 10.1161/01.cir.76.4.860

[7] Romero D, Sebastian R, Bijnens BH, Zimmerman V, Boyle PM, et al (2010) Effects of the purkinje system and cardiac geometry on biventricular pacing: a model study. Annals of biomedical engineering 38: 1388–98. 20094915 10.1007/s10439-010-9926-4

[8] Boyle PM, Massé S, Nanthakumar K, Vigmond EJ (2013) Transmural IK(ATP) heterogeneity as a determinant of activation rate gradient during early ventricular _brillation: Mechanistic insights from rabbit ventricular models. Heart rhythm : the o_cial journal of the Heart Rhythm Society 10: 1710–1717. 10.1016/j.hrthm.2013.08.010 PMC3826179 23948344

[9] Boyle PM, Veenhuyzen GD, Vigmond EJ (2013) Fusion during entrainment of orthodromic reciprocating tachycardia is enhanced for basal pacing sites but diminished when pacing near Purkinje system end points. Heart Rhythm 10: 444–451. 23207137 10.1016/j.hrthm.2012.11.021 PMC3587662

[10] Boyle PM, Deo M, Plank G, Vigmond EJ (2010) Purkinje-mediated effects in the response of quiescent ventricles to defibrillation shocks. Annals of biomedical engineering 38: 456–68. 19876737 10.1007/s10439-009-9829-4

[11] Deo M, Boyle PM, Kim AM, Vigmond EJ (2010) Arrhythmogenesis by single ectopic beats originating in the Purkinje system. Am J Physiol Heart Circ Physiol 299: H1002–11. 20622103 10.1152/ajpheart.01237.2009 PMC3774322

[12] Ijiri T, Ashihara T, Yamaguchi T, Takayama K, Igarashi T, et al (2008) A procedural method for modeling the purkinje fibers of the heart. The journal of physiological sciences : JPS 58: 481–6. 18926006 10.2170/physiolsci.RP003208

[13] Vigmond EJ, Clements C (2007) Construction of a computer model to investigate sawtooth effects in the Purkinje system. IEEE transactions on bio-medical engineering 54: 389–99. 17355050 10.1109/TBME.2006.888817

[14] Atkinson A, Inada S, Li J, Tellez JO, Yanni J, et al (2011) Anatomical and molecular mapping of the left and right ventricular His-Purkinje conduction networks. Journal of molecular and cellular cardiology 51: 689–701. 21741388 10.1016/j.yjmcc.2011.05.020

[15] Tranum-Jensen J, Wilde AA, Vermeulen JT, Janse MJ (1991) Morphology of electrophysiologi-cally identified junctions between Purkinje fibers and ventricular muscle in rabbit and pig hearts. Circulation Research 69: 429–437. 1860183 10.1161/01.res.69.2.429

[16] Mendez C, Mueller WJ, Urguiaga X (1970) Propagation of Impulses across the Purkinje Fiber-Muscle Junctions in the Dog Heart. Circ Res 26: 135–150. 5412530 10.1161/01.res.26.2.135

[17] Wiedmann RT, Tan RC, Joyner RW (1996) Discontinuous conduction at Purkinje-ventricular muscle junction. The American journal of physiology 271: H1507–16. 8897946 10.1152/ajpheart.1996.271.4.H1507

[18] Deo M, Boyle P, Plank G, Vigmond E (2009) Arrhythmogenic mechanisms of the Purkinje system during electric shocks: a modeling study. Heart rhythm 6: 1782–9. 19959130 10.1016/j.hrthm.2009.08.023 PMC5381712

[19] Vigmond EJ, Aguel F, Trayanova NA (2002) Computational techniques for solving the bidomain equations in three dimensions. Biomedical Engineering, IEEE Transactions on 49: 1260–1269. 10.1109/TBME.2002.804597 12450356

[20] Vigmond EJ, Hughes M, Plank G, Leon LJ (2003) Computational tools for modeling electrical activity in cardiac tissue. Journal of electrocardiology 36: 69–74. 14716595 10.1016/j.jelectrocard.2003.09.017

[21] Mahajan A, Sato D, Shiferaw Y, Baher A, Xie LH, et al (2008) Modifying L-type calcium current kinetics: consequences for cardiac excitation and arrhythmia dynamics. Biophysical journal 94: 411–23. 18160661 10.1529/biophysj.106.98590 PMC2157257

[22] Aslanidi OV, Sleiman RN, Boyett MR, Hancox JC, Zhang H (2010) Ionic mechanisms for electrical heterogeneity between rabbit Purkinje fiber and ventricular cells. Biophysical journal 98: 2420–31. 20513385 10.1016/j.bpj.2010.02.033 PMC2877343

[23] Krassowska W (1995) Effects of electroporation on transmembrane potential induced by defibrillation shocks. Pacing Clin Electrophysiol 18: 1644–1660. 7491308 10.1111/j.1540-8159.1995.tb06986.x

[24] Ashihara T, Trayanova NA (2005) Cell and tissue responses to electric shocks. Europace 7 Suppl 2155–165. 16102513 10.1016/j.eupc.2005.03.020

[25] Bishop MJ, Vigmond E, Plank G (2011) Cardiac bidomain bath-loading effects during arrhythmias: interaction with anatomical heterogeneity. Biophysical journal 101: 2871–81. 22208185 10.1016/j.bpj.2011.10.052 PMC3244060

[26] Huizar JF, Warren MD, Shvedko AG, Kalifa J, Moreno J, et al (2007) Three distinct phases of VF during global ischemia in the isolated blood-perfused pig heart. American journal of physiology Heart and circulatory physiology 293: 1617–28. 10.1152/ajpheart.00130.2007 17545483

[27] Azarov JE, Shmakov DN, Vityazev Va, Roshchevskaya IM, Roshchevsky MP (2007) Activation and repolarization patterns in the ventricular epicardium under sinus rhythm in frog and rabbit hearts. Comparative biochemistry and physiology Part A, Molecular & integrative physiology 146: 310–6. 10.1016/j.cbpa.2006.10.036 17188010

[28] Bordas R, Gillow K, Lou Q, Efimov IR, Gavaghan D, et al (2011) Rabbit-specific ventricular model of cardiac electrophysiological function including specialized conduction system. Progress in biophysics and molecular biology 107: 90–100. 21672547 10.1016/j.pbiomolbio.2011.05.002 PMC3190654

[29] Sebastian R, Zimmerman V, Romero D, Sanchez-Quintana D, Frangi AF (2013) Characterization and modeling of the peripheral cardiac conduction system. IEEE transactions on medical imaging 32: 45–55. 23047864 10.1109/TMI.2012.2221474

[30] Abboud S, Berenfeld O, Sadeh D (1991) Simulation of high-resolution QRS complex using a ventricular model with a fractal conduction system. Effects of ischemia on high-frequency QRS potentials. Circulation Research 68: 1751–1760. 2036723 10.1161/01.res.68.6.1751

[31] Panfilov AV (2006) Is heart size a factor in ventricular fibrillation? Or how close are rabbit and human hearts? Heart rhythm : the official journal of the Heart Rhythm Society 3: 862–4. 10.1016/j.hrthm.2005.12.022 16818223

[32] Bishop MJ, Plank G (2012) The role of fine-scale anatomical structure in the dynamics of reentry in computational models of the rabbit ventricles. The Journal of physiology 590: 4515–35. 22753546 10.1113/jphysiol.2012.229062 PMC3467803

[33] Maron BJ, Maron MS (2013) Hypertrophic cardiomyopathy. Lancet 381: 242–55. 22874472 10.1016/S0140-6736(12)60397-3

[34] Joyner RW, Overholt ED, Overholt D (1985) Effects of octanol on canine subendocardial Purkinje-to-ventricular transmission Effects of octanol on canine subendocardial transmission. 249: H122–H1231. 10.1152/ajpheart.1985.249.6.H1228 3000199

[35] Vassall-Adams PR (1983) Ultrastructure of the human atrioventricular conduction tissues. Euro-pean Heart Journal 4: 449–460. 10.1093/oxfordjournals.eurheartj.a061501 6628421

